# Anthocyanins from Agro-Industrial Food Waste: Geographical Approach and Methods of Recovery—A Review

**DOI:** 10.3390/plants12010074

**Published:** 2022-12-23

**Authors:** Zoriţa Diaconeasa, Cristian I. Iuhas, Huseyin Ayvaz, Mustafa Mortas, Anca Farcaş, Mihaela Mihai, Corina Danciu, Andreea Stanilă

**Affiliations:** 1 Faculty of Food Science and Technology, University of Agricultural Sciences and Veterinary Medicine, 3-5 Calea Mănă¸stur, 400372 Cluj-Napoca, Romania; 2 BioTech Technology Transfer Center, 400372 Cluj-Napoca, Romania; 3 Faculty of Medicine, Iuliu Hatieganu University of Medicine and Pharmacy, 400372 Cluj-Napoca, Romania; 4 Department of Food Engineering, Faculty of Engineering, Canakkale Onsekiz Mart University, Canakkale 17100, Turkey; 5 Food Engineering Department, Faculty of Engineering, Ondokuz Mayıs University, Samsun 55139, Turkey; 6 Department of Transversal Competencies, University of Agricultural Sciences and Veterinary Medicine, 3-5 Calea Mănă¸stur, 400372 Cluj-Napoca, Romania; 7 Department of Pharmacognosy, Victor Babes University of Medicine and Pharmacy, 2 Eftimie Murgu Sq., 300041 Timisoara, Romania

**Keywords:** anthocyanins, food waste, bioactive compounds

## Abstract

Drastic growth in the amount of global food waste produced is observed every year, not only due to incessant population growth but also economic growth, lifestyle, and diet changes. As a result of their increasing health awareness, people are focusing more on healthy diets rich in fruits and vegetables. Thus, following worldwide fruit and vegetable consumption and their processing in various industries (juice, jams, wines, preserves), significant quantities of agro-industrial waste are produced (pomace, peels, seeds) that still contain high concentrations of bioactive compounds. Among bioactive compounds, anthocyanins have an important place, with their multiple beneficial effects on health; therefore, their extraction and recovery from food waste have become a topic of interest in recent years. Accordingly, this review aims to summarize the primary sources of anthocyanins from food waste and the novel eco-friendly extraction methods, such as pulsed electric field extraction, enzyme-assisted extraction, supercritical fluid extraction, pressurized liquid extraction, microwave-assisted extraction, and ultrasonic-assisted extraction. The advantages and disadvantages of these techniques will also be covered to encourage future studies and opportunities focusing on improving these extraction techniques.

## 1. About Food Waste

With continuous population growth, the amount of food required also increases, leading to simultaneous rises in food waste, intensifying pollution and economic losses, eventually reaching a stage where waste storage is no longer feasible [1,2,3]. These current population and consumption trends are expected to continue to drive up the food demand for at least another 40 years. Therefore, increasing food supplies and reducing food losses and waste are highly critical in meeting these requirements [4]. For instance, the global population is expected to exceed 9 billion by 2050, needing a 60–70% increase in food production [5]. More precisely, this increase could be met by increasing agricultural land use to 70% of the land area, which is challenging to achieve. Farmers are forced to constantly expand their agricultural land area, or undertake genetic modification techniques to increase production per unit [4]. Thus, the high demand for energy and food essential to meet the needs of society, along with the process of rapid urbanization and the low degree of development of waste management, will determine the accumulation of food waste [6]. In both developed and developing countries, food loss and waste are not only environmental issues, but also social and financial problems [7]. However, what exactly do food loss and food waste mean? There are numerous definitions for these terms, but they are all essentially the same, as the definitions for these two terms will be provided below.

**Figure 1:**
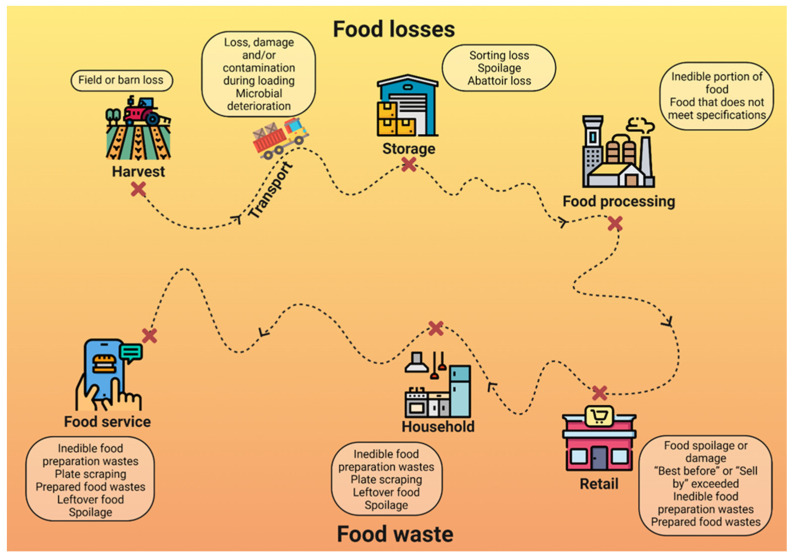
The food chain supply stages and reasons for food waste/loss occurrence at every stage [10].

According to the Food and Agriculture Organization (FAO) of the United Nations, the concept of food loss is “a decrease in the quality or quantity of food.” This includes the term food waste, which refers to the “disposal or misuse (other than food) of food that meets certain safety criteria and is intended for human consumption.” This food waste can occur at any stage along the entire food supply chain, from primary production to the consumer [8]. In Figure 1, the food chain supply stages are shown alongside the reasons behind food waste/loss occurrence at every stage.

One third of the food produced annually goes into the garbage, costing about 1 trillion USD [4]. Such a significant amount of food waste has social and environmental consequences, including social structure issues, land overexploitation, economic problems, food security concerns, the greenhouse effect, and unbalanced global food distribution [4]. Similarly, a study estimated that more than 1.3 billion tons of food are wasted along the entire supply chain from agriculture to consumption by the customer [8]. To be more exact, 413 million tons are lost in the agricultural production stage, 293 million tons are scattered in the post-harvest handling and storage stages, 148 million tons are lost in the processing phase, and 280 million tons are lost at the end of this food chain through household consumption [9].

According to the United Nations Environment Program Publications report on total food waste, over 931 million tons were wasted in the final stages of the supply chain. Based on the report, the most considerable food losses occur in households, accounting for 61% of the total waste, followed by food services at 26% and retail, where waste is 13% [8]. Additionally, the assessment of food waste according to food type shows that the highest percentage of food waste occurs in the case of fruits and vegetables (39–44%), followed by roots and tubers by 33%, then seafood by 24%, and finally 20–22% of all cereals produced. However, these percentages are relative because the amount of waste produced at the household level varies significantly from country to country. The factors influencing this variation are population income and a country’s development and industrialization level [11]. Still, the major groups of food wasted, including fruits and vegetables, constitute a significant source of anthocyanins, implicitly and their residues. Thus, anthocyanins recovery through innovative processes is paramount in the current food waste context.

Considering various data reported on food waste, a considerable amount of waste was produced from the group of fruits and vegetables globally; 25% in Africa, 48% in the United States and Asia, and 53% in Europe. This category is the primary source of anthocyanins. Unfortunately, the existing studies do not mention exact quantities of food waste, only the percentages presented, therefore, these percentages cannot be compared because they relate to different amounts of food consumed and the number of inhabitants, but also to different monitoring factors.

Anthocyanins are one of the many value-added compounds that can be recovered from the agro-industrial wastes of fruits and vegetables that have drawn people’s attention. Despite having significant technological, practical, and financial potential, anthocyanins are still rarely used as food additives in food systems due to the complex process of obtaining them from waste. As a result, selecting an extraction method and setting up extraction conditions are crucial steps in recovering and purifying anthocyanins for use in food [12].

The recovery of bioactive compounds from food loss can be easily achieved with some improvements of existing methods, promoting their economic value through their use in the agri-food and feed industries. In this respect, strategies are needed to protect the environment and educate the consumer about the massive problem of food waste, as well as for smart, sustainable, and inclusive growth [13]. In recent years, these terms have gained more momentum for the population, and it has been acknowledged that food waste substantially impacts the environment and affects global food security [14].

## 2. The Most Food Waste-Producing Substitute Industries

Many industries produce a significant amount of waste; the principal industries contributing to this worldwide issue will be detailed below.

Although these residues are no longer useful to producers, they still contain a significant number of bioactive compounds, which have nutritional and chemical value. These compounds that remain in the natural matrix include proteins, carbohydrates, antioxidants, pectin, vitamins, and phenolic compounds with beneficial effects on human health. Thereby, even if recovering this agro-industrial waste is challenging, it is critical to return them to industrial chains so that they can be used as main food ingredients with potential health benefits [15]. This topic is relevant to the development of functional foods and medicines to treat acute and chronic disorders, as well as antioxidants to be used in the cosmetics sector [16].

According to the FAO, food loss and waste in the fruit and vegetable industries account for 60% of total production [16]. This fruit processing industry produces a wide range of foods such as jams, juices, snacks, or salads, but these processes leave significant amounts of agro-industrial waste such as seeds, peels, and pomace [15]. Juice production residue is the fifth-largest contributor to overall yearly food waste in Europe, accounting for about 3% of the total weight discarded [17].

For instance, roughly 767 MT of apples are produced worldwide annually, and apple pomace generation contributes to around 25–30% of total biomass [18]. The apple processing industry is one of the significant sources of waste production, the most common residue being the peel. Phytochemicals such as anthocyanins, phenolic acids, and flavonoids are present in apple peel with anticancer and cardioprotective properties. Additionally, previous research has reported that apple peel contains roughly 80% of polyphenols and has a higher total antioxidant capacity than apple core [19].

Another specific industry that produces a high amount of waste is the grape processing industry. In 2013, 77.2 million tons of grapes were produced globally, of which 55% were used in the winery industry, with about 20% agro-industrial wastes generated [20]. According to a few studies, red grape marc is an excellent raw material for producing bioactive molecules of industrial importance since it contains a high concentration of phenolic chemicals [21]. Pomace or marc is the outcome of the juice production or vinification process, where grapes are mechanically pressed or fermented, followed by drying the remaining pomace [22].

Therefore, the wine industry, an important but not the sole sector in grape processing, produces 84.9 million tons of waste worldwide. On average, one ton of crushed wine grapes can yield 600 kg of grape pomace on average, in which impressive amounts of flavonoids and anthocyanins can be found [20,21,22,23]. Hence, high amounts of anthocyanins could be extracted from grape marc after vinification, the content of recovered anthocyanins being higher than that of grape peel and even higher than wine. Additionally, they have a higher degree of stability because most of them are acylated or methoxylated [21].

Another industry that generates a significant amount of waste is related to berries. Since berries are one of the most abundant sources of health-promoting phytochemicals, and their cultivation and consumption are rising, implicitly, the increase in their production will lead to a significant amount of waste [23,24]. Berries include bilberries, blueberries, lingonberries, and cranberries, which are important sources of anthocyanins. For example, the average production of blueberries in the United States (2009–2013) was about 200,000 tons [25,26].

Most berries are quite perishable fruits, so large amounts are processed into juices, jams, and purees, leaving a significant amount of berry residue, namely pomace [27]. Berry pomace contains fruit pulps, skins, pulp, stems, and significant amounts of bioactive compounds such as lipids, phenolic compounds, and fiber. Some studies reported that about 18% of the total anthocyanin content in blueberries remains in the pomace and is not extracted with juice pressing. Therefore these compounds can be recovered and used as a source of ingredients in the food, health, and cosmetics industries [27,28,29].

Another fruit that is commonly used in various industries is the plum. Plums are cultivated in most countries worldwide, with a global production of plums and sloes of 12.6 million tons. The region with the highest plum production is Asia, with 8,184,840 tons representing 64.9% of total production, followed by Europe with 3,013,138 tons and America with 903,399 tons. Moreover, it must be stated that the countries with the largest plum production are China, with 6,788,107 tons, and Romania, with 842,132 tons. Plums can be used as a raw material in the food and beverage industries in the production of significant amounts of canned fruits, juices, and alcoholic beverages that are very common in Central and Eastern Europe. Therefore, during the industrial processing of plums, tons of pomace are created, mainly containing the peel and pulp left over from the production of juices, alongside seeds. Distillery waste after obtaining brandy also contains the pulp and peel of the fruit used. These residues contain bioactive compounds such as lipids, carbohydrates, and phenolic compounds with decent antioxidant capacity. Plum peel contains the highest number of phenolic compounds, thus contributing to the antioxidant capacity of plum pomace. The compounds that are found in the highest concentration in plum pomace are flavanols and anthocyanins, which are to be extracted from this waste and used in the future because they are a cheap source of principal substances that could be utilized in various fields, such as cosmetics and pharmaceuticals [30].

In the same category, sweet cherries are some of the most prized and consumed fruits in temperate regions, including Europe, Asia, North Africa, America, and Australia [31]. This is a popular fruit thanks to its flavor, color, nutritional content, bioactive characteristics, and health benefits. The production of cherries is on an upward trend, increasing yearly. Cherries were globally produced by about 2.5 million tons in 2018. Sweet cherries are most often eaten as fresh. However, the cherry processing industry includes various products such as frozen, canned cherries, juices, wine, jams, and alcoholic beverages [32]. The massive volume of processed cherries leads to significant waste, including stems, seeds, and pomace [32,33]. There has been much interest in reusing cherry waste since it could be a source of high-value bioactive chemicals that are currently underutilized. All the wastes mentioned are rich in different bioactive compounds, but a significant amount of anthocyanins is only found in pomace [33]. Like the other fruits, in the case of cherries, the most significant quantities of anthocyanins and other phenolic compounds are found in the peel, compared to the fruit’s flesh. Therefore, the pomace or press cake from sweet cherries still includes a lot of anthocyanins and other phenolics. Consequently, sweet cherry pomace is an important source of anthocyanins with various biological activities such as antioxidant, anticancer, anti-inflammatory, and chemopreventive [32]. Further studies are needed to extract these compounds from pomace, for a higher bioactive valorization from residual biomass.

As observed, the processing techniques of vegetables, fruits, and cereals generate significant amounts of agro-industrial residues, which are frequently classified as waste and thrown away. Some examples of these processing agro-industrial waste include damaged raw materials, peels or skins, seeds, brans, husks, hulls, and cobs, with quantities increasing significantly every year. The significant amount of food waste generated during manufacturing has been studied for the recovery of valuable components with underlying economic and environmental reasons. Among the molecules present in these wastes, anthocyanins particularly stand out due to their coloring properties, as well as their potential positive effects on human health, such as antioxidant, anticancer, anti-inflammatory, and antimicrobial properties [34,35]. Therefore, it is necessary to deepen the methods of extracting anthocyanins from waste and make them more efficient to recover as large and pure anthocyanins as possible.

## 3. Valuable Bioactive Compounds from Food Waste, Particularly Anthocyanins

As already mentioned, several bioactive compounds in the phytochemical composition of agro-industrial waste end up in landfills, but still possess potential benefits for human health. The bioactive compounds in the waste can be phenolic compounds, phenolic acids, alkaloids, terpenoids, fatty acids, peptides, and many others that must be recovered from the residue matrix [21]. Therefore, some of the most important compounds and their associated waste to be extracted will be summarized in the next section, with particular emphasis on anthocyanins.

On the one hand, carotenoids are indispensable compounds in red and orange fruits and vegetables with an essential role in their coloration. Moreover, carotenoids are an essential component of the food field due to their characteristics, such as their antioxidant activity and use as a food colorant. Due to their functions, these compounds are of major interest not only for food but also in other fields such as pharmaceuticals, cosmetics, or chemicals. There are recent studies on carotenoids extracted and recovered from the vegetable and fruit waste matrices, such as grape, tomatoes, and banana peels, pomegranate, and pumpkin seeds, as well as apricot bagasse and pomace [36].

There is also pectin, a natural anionic polysaccharide with a high molecular weight, which is biocompatible, non-toxic, and can be isolated from the cell walls of higher plants. Pectin is a functional ingredient in the food industry, acting as a stabilizer or thickener, a critical compound in manufacturing jams, jellies, fruit juices, and other foods [37]. This compound can be recovered from different types of waste, including apple pomace, with about 14% of world pectin production being extracted from apple pomace or citrus peel waste. Citrus pectin’s usefulness as a valuable nutraceutical compound has also been demonstrated in several research studies, directly affecting immune cells to modulate inflammatory responses [25].

Ultimately, anthocyanins are another valuable group of compounds that can be recovered from food waste. They are a class of natural water-soluble pigments that have a significant role in seed dispersal, pollination, and development of plant organs and adaptation to various changes in biotic and abiotic factors. Numerous studies have reported that anthocyanins have important antioxidant, anti-inflammatory, antimicrobial and anticancer properties and thus can be used in the chemoprevention of various diseases such as diabetes, obesity, and even cancer [35]. Because of these various characteristics of anthocyanins, new studies focus on the recovery of anthocyanins from food waste, as a topical issue, due to the tremendous amount of waste produced globally.

Therefore, food industry residues generated after industrial processing (peels, seeds, pomace, and even leaves) contain high amounts of bioactive compounds such as phenols, carotenoids, fatty acids, anthocyanins, enzymes, and fibers. They can be used to create functional foods, antioxidants in the cosmetics industry, and even for medicines against chronic and acute diseases [16]. Due to their utmost relevance and usefulness, anthocyanins need a more extensive overview. Anthocyanin recovery based on the type of waste extracted and the method type used for the extraction are summarized in Table 1.

## 4. Methods for the Recovery of Bioactive Compounds from Food Waste

Even though the organic material found in agro-industrial waste is a valuable source of components, only a few methods can guarantee the safety of the recovery, purification, and concentration of these bioactive molecules [21]. The source, activity, chemical characteristics, and end-use all influence the extraction of bioactive chemicals from agro-industrial waste [56].

There are widely used methods of extracting bioactive compounds from food waste, including steam distillation, solvent extraction, CO_2_ extraction, and Soxhlet extraction [57]. Even though conventional organic solvent extraction techniques are easy to use, low-cost, and do not require special equipment, they have many disadvantages [58]. These disadvantages include the restricted type and a large amount of solvent required for extraction. The most common solvents used in extractions are volatile, but they are toxic, so in the extraction of compounds from residues, they are unsafe for human consumption and can cause carcinogenic risks in humans. Among other disadvantages of these conventional techniques, we can mention the lengthy extractions and the relatively low recovery efficiency for bioactive compounds from waste [57].

Therefore, in this field of sustainable waste reuse, several innovative technologies are capable of carrying out the processes mentioned above necessary for the recovery of biocomposites. However, future studies will be required to consolidate them and expand their industrial usefulness [21].

In this respect, green extraction methods have emerged as an excellent alternative to extracting high-added value compounds from agro-industrial wastes in this context. These procedures generate a high-quality and pure final extract. Examples of such technologies that apply to these matrices are supercritical fluid extraction (SFE), ultrasonic-assisted extraction (UAE), microwave-assisted extraction (MAE), which are environmentally friendly due to lower energy consumption, use a reduced volume of organic solvents and have a short operating time [21].

The unconventional extraction techniques of anthocyanins applicable to their recovery from food waste are detailed below.

### 4.1. Supercritical Fluid Extraction

Supercritical fluid extraction (SFE) is one of the most sustainable green technologies; and has been extensively employed in recent decades [59]. The principle of operation of this method involves a supercritical fluid as the extraction medium, which is introduced into the substances containing target components, and then the extraction is performed based on the differences in solubility [60]. In brief, there are two main steps, the first is when the desired compound is extracted from a supercritical fluid, and the second is when the liquid is rapidly removed by a change in temperature and pressure [59].

This method uses CO_2_ as the solvent, which is suitable for selectively extracting soluble chemicals from vegetable raw materials [61]. Hence, using this compound as a solvent has some advantages. Firstly, it is non-explosive, readily available, and can be removed from the final extract. Furthermore, it is not expensive, and most importantly, it is not toxic and does not cause bioactive compounds to undergo significant chemical changes, maintaining their biological characteristics [60,61]. The main disadvantage of this method is that the solvent is non-polar, so this technique is more suitable for extracting non-polar pigments, such as carotenoids or chlorophylls, rather than polar ones as betalains and anthocyanins [58]. However, in the case of anthocyanins, a co-solvent is used to promote the extraction of polar compounds. Thus, the most common co-solvents used are ethanol, methanol, and the aqueous solutions of these alcohols in different concentrations. Additionally, in terms of disadvantages, the high cost of this technique should be mentioned. In this respect, further studies are needed to calculate the operational costs of an industrial-scale extraction [59].

As already mentioned, this method can be used to recover anthocyanins from different matrices. The extraction conditions can be optimized to obtain maximum extraction efficiency. The adjustable parameters include pressure, temperature, particle size, amount of co-solvent, moisture content of the natural material, extraction time, CO_2_ flow, and liquid–solid ratio [59].

### 4.2. Ultrasonic-Assisted Extraction

Oscillating sound pressure waves with a frequency of over 20 kHz help accelerate the mass transfer between the solvent and the plant material [62]. Thus, ultrasonic assisted extraction (UAE) is another method based on the cavitation phenomenon, a mechanical process generated by ultrasounds that causes mass transfer and compound extraction at low temperatures and short extraction times [63]. Cavitation exerts mechanical effects on the matrix, improving mass transfer and breaking up the matrix, resulting in increased target compound recovery. In addition, bubbles are created during cavitation that grow to a critical size and then collapse, releasing vast amounts of energy that raise the temperature and pressure of the solvent [64].

UAE can be performed in two ways: directly irradiating the probe or using an ultrasonic bath. The latter is more cost-effective and simpler to use but has some disadvantages as it generally functions at a single frequency, 20 or 40 kHz, and the energy produced is not spread evenly inside, thus limiting extraction efficiency [59,60,61,62,63,64]. Direct ultrasonic extraction consists of a probe with the sample in a transducer immersed in the extraction vessel, where the ultrasound disperses in the environment with a minimum energy loss. This way, a higher ultrasonic intensity is obtained than with the system involving an ultrasonic bath, since the ultrasound energy is concentrated in a specific area of the sample, elevating the extraction efficiency [59].

This extraction method can be implemented on a large scale. However, it is necessary to have specific characteristics such as sustainability, profitability, safety, and being environmentally friendly. These are possible to achieve by intensifying the process and reducing energy consumption. The parameters that influence UAE efficiency are the intensity/amplitude, ultrasonic power, and frequency of the ultrasonic waves [60].

UAE is commonly used to extract bioactive compounds from various natural matrices. Nevertheless, the extraction conditions must be optimized to get the highest antocyanin yield using UAE. The main matrix-specific parameters that can be optimized to increase the extraction efficiency are time, solvent composition, temperature, the liquid–solid ratio, particle size, moisture content in the sample, and ultrasound power [59].

Relevant research on anthocyanins extraction using UAE from purple eggplant peels has revealed that the number of anthocyanins recovered increased with increasing ultrasonication time. The maximum concentration of anthocyanins peaks after 30 min of sonication, about 29.011 mg GAE/g DM (gallic acid equivalents per dry weight) in peel extracts using acidified water as solvent. In addition, the authors identified two anthocyanins, namely malvidin-3-rutinoside-5-glucoside and cyanidin-3-rutinoside, for the first time in eggplant peel extract. Although anthocyanin extraction was enhanced using an ultrasonic probe, microscopic analysis revealed that cell denaturation and damage occurred [65].

### 4.3. Microwave-Assisted Extraction

Microwave-assisted extraction (MAE) uses electromagnetic radiation energy from the microwave region. Microwave absorption excites molecules to upper rotational levels, dissipating this excess energy to the medium in the form of heat. This method relies on rapid heating by microwave radiation, which causes the plant cell walls to break down as the cell structure expands [58]. As a result, the technique is based on focused or non-focused applications on a sample of non-ionizing electromagnetic waves of frequency ranging from 300 MHz to 300 GHz [12]. There are three steps for this type of extraction; the first involves separating the dissolved substances from the sample matrix, then increasing the temperature and pressure, and finally, the solvent diffuses into the sample matrix [44].

MAE can extract bioactive compounds faster and with improved recovery results. Like other methods, specific parameters may change the extraction efficiency. In this case, these parameters are temperature, frequency, irradiation time, solid–liquid ratio, microwave power, extraction pressure, solvent type and composition, and matrix particle size [59,60]. An important parameter in microwave extraction is the type of solvent that absorbs microwave energy. Its ability to solubilize the target compounds and absorb microwave energy is critical for recovering the desired compounds. Therefore, the most commonly used solvents for this type of extraction are those with a high dielectric constant. Hence, polar solvents such as water and ethanol can absorb electromagnetic energy and release it as heat, favoring the extraction [12].

Due to the cell disruption induced by internal warming, MAE technology increases anthocyanin extraction by increasing mass transfer rates. As a result, anthocyanins release into the extraction medium occurs in higher amounts [12].

The advantages of this extraction method mentioned in the existing studies include the shorter time, the reduced amount of solvent used, the increased yield compared to a conventional extraction, and better energy saving due to its quick processing time. However, a disadvantage of this method is the negative effect of microwave energy on anthocyanins in the sense that the intensification of microwaves leads to a decrease in the extraction efficiency of anthocyanins due to their degradation [46,59].

### 4.4. Pulsed Electric Field

Pulsed electric field (PEF) is a non-thermal method that uses direct current; thus, the high voltage pulses will pass through the plant material placed between the electrodes in a brief time interval of ordinal milliseconds [66]. This technique is based on the passage of electric potential through the membranes of living cells, which separates depending on the nature of the dipole and the charge of the membrane molecules [60]. The induced electric field can cause the development of hydrophilic pores in the cell membrane, which allows protein channels to open. In this way, the electroporation process takes place, meaning the formation of temporary (reversible) or permanent (irreversible) pores, which will increase the permeability of the cell [12,59]. Therefore, PEF treatment improves the migration of target chemicals in the cytoplasm across the cell membrane, resulting in increased mass transfer, higher extraction rates, and yields [12]. Shortly, the membrane cell loses its structural functioning, making the extraction of bioactive chemicals easier [59]. The extraction efficiency of target compounds by PEF depends on several parameters such as temperature, treatment period, electric field strength, conductivity, pH and ionic strength of the medium, specific energy input, and pulse number [60,67].

Among the advantages of this extraction method, one could include the lack of adding chemicals, thus reducing the operational cost and making it a sustainable method. At the same time, pulsed electric field extraction does not require heat, thus using less energy and making this method feasible for the extraction of heat-sensitive compounds, such as anthocyanins [60]. However, some studies mention that this method is more expensive than thermal techniques, and the samples subjected to this type of extraction must have their electrical conductivity reduced and be free of air bubbles [59,60]. Moreover, applying a current of too high voltage can cause the degradation of anthocyanins into chalcone and other pseudobases. Hence, for maximum anthocyanin extraction yield, the best PEF treatment is the one that uses low or moderate intensity, thus avoiding their degradation [12,68].

In recent years, most of the research on the subject of PEF has highlighted the advantages of its application in the recovery of bioactive molecules from agro-industrial waste. However, some gaps still exist, and further experimental studies are needed [67].

### 4.5. Enzyme-Assisted Extraction

Currently, enzyme-assisted extraction (EAE) of bioactive compounds is gaining popularity as a viable alternative to traditional solvent extraction methods. EAE is a cost-effective and environmentally-friendly extraction approach based on the property of enzymes to catalyze reactions with high specificity, and regioselectivity, alongside their ability to produce reactions under mild conditions, meaning low-temperature levels and short time intervals [69,70]. In this type of extraction, enzymes can disrupt the cell wall’s structural complexity and hydrolyze its components [71]. Thus, the cell wall is disintegrated, and the cell permeability increases while intracellular components are released [69,71]. This breakdown allows the solvent, which can be water or organic, to penetrate and aid in metabolite [72].

Targeted compounds are often stored in plant cells, so more barriers must be crossed to reach them. Obstacles are extracellular cell walls, cell walls, and oleosomes, but they can be hydrolyzed naturally by specific enzymes. Therefore, the most used enzymes for the hydrolysis of plant cell wall compounds are of 4 different types: hemicelluloses, cellulases, proteases, and pectinases. Each of these enzymes can perform a selective extraction, alone or in combination, of the bioactive compounds present in the sample [59].

Compared to traditional methods, EAE methods have shown lower energy consumption, faster extraction rates, higher extraction yield, and more straightforward recovery with less solvent utilization [69]. Nevertheless, EAE also has certain disadvantages, including the high price of the enzymes used, which limits the use of this method only for high-value compounds. At the same time, the enzymes used cannot destroy the cell matrix. Thus, a further step of separating and purifying the sample is required. In addition, the enzymes have poor recyclability and reusability, losing their catalytic activity after being used several times. Therefore, these disadvantages make EAE a high-cost technique and render it unfeasible for large-scale industrial use [72].

Parameters that can be modified for better EAE efficiency are pH and system temperature, enzyme concentration and composition, extraction time, liquid–solid ratio, and particle size of the substrate [71]. Thus, by modifying these operating conditions, this method can achieve a high yield compared to conventional techniques in extracting various plant compounds such as phenols, pigments, and anthocyanins from different natural matrices. Therefore, one of the major advantages of this method is that an enzymatic mixture can be prepared depending on the type of matrix and the compound to have the highest possible yield [59].

### 4.6. Pressurized Liquid Extraction

Another extraction technique is pressurized liquid extraction (PLE), which is also called accelerated solvent extraction (ASE), pressurized fluid extraction (PFE), enhanced solvent extraction (ESE), or high-pressure solvent extraction (HSPE) [60]. The principle of this method is based on applying high pressure to keep the solvent liquid at very high temperatures above its boiling point, thus improving the solubility of the compounds, wetting the sample, and penetrating the matrix [68]. These conditions cause a low surface tension and viscosity, thus favoring the penetration of the solvent into the solid matrix and producing an increase in the rate of mass transfer and diffusion. At the same time, the presence of air in the vacuoles of the plant cells produces a high-pressure leakage, which causes the denaturation of cell membrane proteins, thus increasing the accessibility to the desired compounds during extraction [73].

The pressure in this type of extraction varies from 100 MPa to values above 1000 MPa, ensuring better penetration of the solvent in the cell membrane to increase bio-accessibility. Based on the literature, the higher the hydrostatic pressure, the more cellular components can be released, and the extraction efficiency will increase, while it can lead to a decrease in selectivity [59,74].

An advantage of liquid extraction under pressure is the possibility of using different solvents, thus recovering both polar and non-polar compounds. Hence, solvents considered eco-friendly can be used, such as ethanol, ethyl lactate, ethyl acetate, and even water [75]. Other advantages of this method are the use of a lower volume of solvent, a shorter extraction time, and the increased bio-accessibility of the bioactive compounds present in the sample. However, the cost of the energy required to produce higher pressures is the principal downside of these extraction technologies [59].

Specific polyphenol families, such as phenolic acids, flavonols, anthocyanins, and stilbenes, can be recovered by adjusting the extraction conditions [76,77]. Among the parameters that can be modified are pressure, the composition of the solvent, particle size, temperature, time of extraction, liquid–solid ratio, and time of extraction [59]. This guarantees its overall effectiveness for extracting phytochemical components from vegetables and fruit waste, such as peels [77].

All the methods presented above are suitable for anthocyanin extraction; however, as it was already mentioned, they have advantages and disadvantages, as summarized in Table 2.

## 5. Conclusions and Future Perspectives

The present review aims to gather scientific information about the current status of global food waste, a significant issue nowadays, and bioactive compounds that can be recovered from food waste through novel green extraction techniques.

Agro-industrial waste is generated after processing, and various products derived from fruits, vegetables, and cereals, are serious threats to the environment on many levels. However, the main hazards are greenhouse gases, the main factors of global warming. Notably, although they are considered waste, these residues may contain large amounts of bioactive compounds such as phenolic acids, fatty acids, anthocyanin, peptides, and many others.

It is for this reason that the trend for sustainability and circular bioeconomy in recent years has focused on the recovery of these valuable compounds through extraction methods that are as environmentally friendly as possible and their use in various industries, such as the food industry, in order to increase the nutritional value of certain foods. There are currently several new extraction methods that are feasible for the extraction of anthocyanins, such as enzyme-assisted extraction, pressurized liquid extraction, supercritical fluid extraction, ultrasonic-assisted extraction, microwave-assisted extraction, and pulsed electric field. Each exhibit both advantages and disadvantages, with the main drawback being that most of them cannot be applied on an industrial scale. Therefore, future studies are needed, not only for optimizing these methods but also for the invention of new extraction methods.

Future perspectives in the recovery and usage of anthocyanins focus on the valorification of these compounds in different fields. There are numerous studies about such potential applications of anthocyanins recovered from waste, such as the production of colorimetric indicator films for monitoring the freshness of food in the packaging sector [78], as colorants and antioxidants agents in the dairy industry [12], as natural pigments in pharmaceuticals and cosmeceuticals industry [79], and manufacturing of dietary supplements or nutraceuticals [80].

## Figures and Tables

**Table 1:** Waste rich in anthocyanins, their quantification, major compounds determined, and method of extraction employed.

Source	Waste	Quantity of ACN Recovered (C3G mg/kg)	Major Compound	Method of Extraction	Refs.
Apple	Peels	21.1		Mixture of HCl/ methanol	[38]
Grapes	Pomace	7419		Pressurized liquid extraction with ethanol	[39]
Sweet cherry	Pomace	4850	Cyanidin-3-glucosyl-rutinoside	The mixture of HCl/methanol/water ultrasound-assisted extraction	[40]
Sour cherry	Pomace	38.20 mg/L		Ultrasonic extraction	[41]
Blueberries	Pomace	1258.2	Delphinidin-3-glucoside	The mixture of HCl/methanol/water	[42,43]
Blackberries	Pomace	1924.2	Cyanidin-3-O-glucoside	The mixture of HCl/methanol	[44]
Chokeberries	Pomace	48,600	Cyanidin-3-O-galactoside	Ultrasound-assisted pressurized liquid extraction	[45]
Raspberries	Pomace	1880.5	Cyanidin-3-O-sophoroside	The mixture of HCl/methanol	[44]
Plums	Skin	24,381		Drying	[46]
Eggplant	Peels	2275	Delphinidin-3-coumaroylrutinoside-5-glucoside	Extraction with methanol, then ultrasonic treatment 45 kHz, 50 °C for 50 min	[47]
Red cabbage	Pomace	546 mg/L		Ultrasonic extraction	[41]
Purple corn	Cob	19,770	Cyanidin-3-(6″malonyl) glucoside	Microwave-assisted extraction	[48]
Blackcurrant	Pomace	25,497.5	Cyanidin-3-O-rutinoside Delphinidin-3-rutinoside, Delphinidin-3-O-glucoside	Microwave vacuum drying	[49]
Redcurrant	Pomace	1499.1	Cyanidin-3-O-sambubioside	Mixture of HCl/methanol	[44]
Cranberry	Pomace	1214	Cyanidin-3-O-galactoside Peonidin-3-O-galactoside	Extrusion and solvent (acetone/ water/acetic acid)	[50]
Mulberry	Pomace	2855	Cyanidin-3-glucoside Cyanidin-3-rutinoside	Boiling with 3% sodium hexametaphosphate and acetone	[51]
Strawberry	Pomace	340	Pelargonidin-3-glucoside	80% methanol aqueous solution with 0.05% acetic acid	[52]
Purple potato	Peels	2935.7	Petunidin	The mixture of HCl/methanol and ultrasonic treatment 45 °C, 100 W for 30 min	[53]
Black carrot	Pomace	1889.5 mg/L		Microwave heating	[54]
Red onion	Skins	7544		Conventional extraction with ethanol	[55]

**Table 2:** Comparison of advantages and disadvantages of novel extraction method.

Extraction Method	Advantages	Disadvantages	Refs.
SFE	Uses a green and renewable solvent	Expensive technique	[72,73,74]
UAE	Short period of extraction Small amounts of solvent	The large-scale application is limited due to high cost	[59,60,61,62,63]
MAE	Fast and efficient technique	Increased technical difficulty	[77]
PEF	Short period of extraction	Degradation of certain compounds can occur from high electric field	[59]
EAE	High selectivity because of the enzymes’ specificity	Expensive cost of enzymes	[59]
PLE	Short period of extraction Suitable for phytochemical extraction	High cost of equipment	[59]

## Abbreviations

Anthocyanins	ACNs
Supercritical fluid extraction	SFE
Ultrasonic assisted extraction	UAE
Microwave-assisted extraction	MAE
Pulsed electric field	PEF
Enzyme-assisted extraction	EAE
Pressurized liquid extraction	PLE
